# Late-Onset En Coup de Sabre: A Rare Presentation of Linear Scleroderma in an Elderly Woman

**DOI:** 10.7759/cureus.109110

**Published:** 2026-05-18

**Authors:** Noor Jasim, Farah Farooqui, Mohammed K Shariff, Farrookh Haider, Khalid Farooqui

**Affiliations:** 1 Internal Medicine, Hamad Medical Corporation, Doha, QAT; 2 Medicine and Surgery, Raha Medical Center, Al Khor, QAT; 3 Cardiology, Hamad Medical Corporation, Doha, QAT

**Keywords:** autoimmune skin disease, en coup de sabre, late onset morphea, linear scleroderma, localized scleroderma

## Abstract

En coup de sabre (ECDS) is a rare form of linear scleroderma that typically presents in childhood and may lead to progressive craniofacial tissue atrophy. Adult-onset disease is uncommon and may delay recognition. Early identification is important to prevent permanent deformity and potential neurologic complications. A 60-year-old woman with no significant past medical history presented with a nine-month history of progressive hyperpigmentation, skin tightening, and indentation involving the nasal bridge and left frontoparietal region. Over time, the discoloration extended toward the right forehead and was associated with contour changes of the nasal bridge and left forehead. Laboratory evaluation revealed a positive anti-histone antibody, with no evidence of systemic autoimmune or infectious disease. Imaging and endoscopic evaluation excluded sinonasal, bony, and intracranial pathology. Skin biopsy findings were consistent with a localized sclerosing process. A diagnosis of linear scleroderma ECDS was established. The patient was initiated on systemic corticosteroid therapy along with topical treatment and referred for multidisciplinary management. This case highlights an atypical late-onset presentation of ECDS. Although most cases occur in pediatric populations, adult-onset disease can present diagnostic challenges and may be associated with delayed recognition. The potential for neurologic involvement, even in the absence of initial symptoms, underscores the importance of careful evaluation and monitoring. Linear scleroderma ECDS should be considered in adults presenting with progressive craniofacial atrophy. Early diagnosis, prompt initiation of therapy, and ongoing neurologic surveillance are essential to reduce the risk of long-term functional and cosmetic sequelae.

## Introduction

Localized scleroderma, or morphea, is a rare inflammatory fibrosing disorder that affects the skin and underlying soft tissues, typically without systemic organ involvement [1,2]. Its incidence is estimated at two to three cases per 100,000 persons per year, and it is more common in females. Among its clinical variants, linear scleroderma is particularly associated with childhood onset and may extend into deeper structures, including subcutaneous fat, muscle, and bone, potentially leading to functional deficit and cosmetic deformity [1,2].

A subtype of linear scleroderma that involves the face and scalp is known as en coup de sabre (ECDS). It manifests as a unilateral, indurated linear band over the frontoparietal region, resembling the strike of a sword, and can be associated with extracutaneous complications such as neurologic, ocular, and musculoskeletal manifestations. While it predominantly presents during the first two decades of life, cases in adulthood are uncommon and may pose diagnostic challenges. Early recognition and timely immunosuppressive therapy are essential to prevent progression and permanent disfigurement [1,2]. We present a rare case of ECDS in an elderly female, highlighting the importance of considering this diagnosis even in atypical age groups.

## Case presentation

Case history

A 60-year-old woman with no significant past medical or family history presented with a nine-month history of progressively worsening lesions over the central face and forehead. The condition initially began as a small, pruritic, hyperpigmented macule on the nasal bridge, which gradually enlarged and extended inferiorly to involve the nasal septum and superiorly toward the left forehead. Over the subsequent two months, hyperpigmentation continued to spread linearly across the left frontoparietal region and then progressed contralaterally across the midline toward the right forehead.

Simultaneously, the patient noticed a gradual sharpening of the nasal bridge contour and indentation of the left frontal region, resulting in visible asymmetry of the face. She denied pain, dysesthesia, ulceration, sensory loss, epistaxis, or nasal obstruction. She also denied alopecia but reported thinning and partial loss of hair over the lateral third of the eyebrows. There was no history of preceding trauma, insect bite, topical cosmetic/whitening cream use, or herbal product application. She reported no preceding infections. Review of systems was negative for fever, weight loss, chronic cough, breathlessness, headache, seizure, visual symptoms, joint pains, gastrointestinal symptoms, or symptoms suggestive of Raynaud phenomenon.

Physical examination

**Figure 1:**
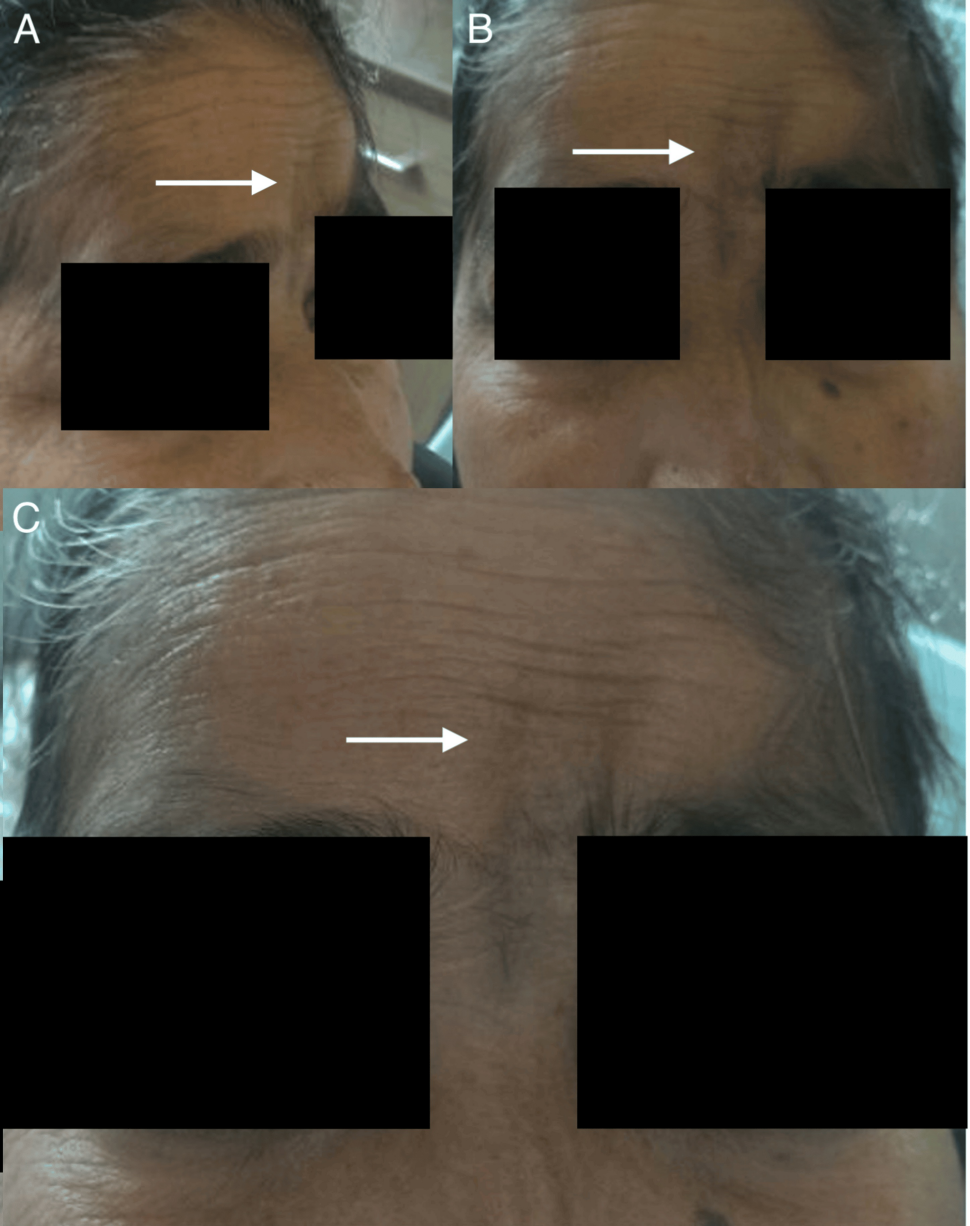
(A) Lateral view showing linear induration over the left frontoparietal region (arrow). (B) Frontal view demonstrating hyperpigmented, atrophic band across the forehead (arrow). (C) Close-up view highlighting central depression and skin atrophy (arrow).

On examination, vital signs were within normal limits. Dermatologic evaluation revealed a linear, hyperpigmented, and sclerotic band extending vertically from the glabella down along the nasal bridge (Figure 1).

The involved skin appeared atrophic, indurated, and depressed relative to the surrounding tissue, with reduced pliability and blunting of normal skin creases. There was noticeable asymmetry of the upper face due to soft-tissue volume loss over the left frontonasal region. Partial thinning of the lateral third of both eyebrows was noted, indicating adnexal involvement. Mild peripheral erythema was present; however, no ulceration, telangiectasia, or scalp alopecia was appreciated. Skin tightening was also noted over the dorsum of the hands without sclerodactyly, nail fold changes, or digital ulcers. Neurologic, musculoskeletal, cardiopulmonary, and abdominal examinations were unremarkable.

Differential diagnosis

Given the progressive linear cutaneous involvement with pigmentation and soft-tissue atrophy affecting the nasal and frontal areas, initial working differentials included guttate morphea, atrophic rhinitis, deep-seated fungal infection, vasculitis, and Hansen’s disease.

Laboratory investigations and imaging

Laboratory investigations are summarized in Tables 1, 2 and were overall unremarkable except for a positive anti-histone antibody. These results ruled out systemic sclerosis, active vasculitis, and infectious etiologies such as syphilis or HIV-related dermatoses. 

**Table 1:** Quantitative laboratory investigations at presentation.

Test	Result	Reference range
Hemoglobin	12.9 g/dL	12.0–16.0 g/dL
White blood cells	6.2 × 10⁹/L	4.0–11.0 × 10⁹/L
Platelets	231 × 10⁹/L	150–400 × 10⁹/L
Absolute eosinophil count	200 cells/µL	0–500 cells/µL
Erythrocyte sedimentation rate (ESR)	12 mm/h	0–20 mm/h
Calcium	9.1 mg/dL	8.5–10.5 mg/dL
Alkaline phosphatase	88 IU/L	44–147 IU/L
Thyroid-stimulating hormone (TSH)	2.45 mIU/L	0.4–4.0 mIU/L

**Table 2:** Qualitative serologic and autoimmune investigations at presentation. ANA: antinuclear antibody, Scl-70: scleroderma 70, C-ANCA: cytoplasmic antineutrophil cytoplasmic antibody, P-ANCA: perinuclear antineutrophil cytoplasmic antibody, HIV: human immunodeficiency virus, VDRL: Venereal Disease Research Laboratory test.

Test	Result	Reference range
Antinuclear antibody (ANA)	Negative	Negative
Anti-topoisomerase I (Scl-70)	Negative	Negative
Rheumatoid factor	Negative	Negative
C-ANCA	Negative	Negative
P-ANCA	Negative	Negative
HIV serology	Non-reactive	Non-reactive
VDRL	Non-reactive	Non-reactive

Chest X-ray (PA view) showed no abnormality. Contrast-enhanced CT of the paranasal sinuses and head revealed no sinonasal mucosal thickening, bony erosion, intracranial extension, or skull bone involvement. Fiber-optic nasopharyngoscopy confirmed intact mucosa without crusting, perforation, ulceration, or discharge. Magnetic resonance imaging (MRI) of the brain was not performed at the time of evaluation because the patient did not exhibit neurological symptoms or focal neurological deficits, and the patient declined further advanced imaging due to financial constraints.

Histopathology

A skin biopsy taken from the affected frontal plaque demonstrated dermal thickening with dense collagen deposition, reduced adnexal structures, and absence of eosinophilic infiltrates, granulomas, or necrosis. No fungal elements were identified on routine hematoxylin-eosin staining. Histopathological slides were reviewed at the time of diagnosis; however, the original slides were subsequently taken by the patient when she returned to her home country, and therefore, histopathological images were not available for inclusion in this report.

Final diagnosis, treatment, and outcomes

Based on the progressive linear induration involving the frontoparietal region, associated soft-tissue atrophy, exclusion of infectious and vasculitic causes, and supportive histopathology, a diagnosis of linear scleroderma with ECDS subtype was established. The patient was commenced on systemic corticosteroids at 1 mg/kg/day, alongside topical corticosteroids to alleviate local dryness and pruritus. She was referred to Dermatology and Rheumatology services for multidisciplinary management, including consideration of photochemotherapy (PUVA therapy) and initiation of a steroid-sparing immunosuppressive agent. Ongoing follow-up was arranged to monitor clinical progression and treatment response. Shortly after the initial evaluation, the patient returned to her home country and was subsequently lost to follow-up at our institution. She had previously indicated her intention to continue care locally in her home country. Consequently, long-term treatment response and disease progression could not be assessed.

## Discussion

ECDS is a craniofacial subtype of linear localized scleroderma characterized by a linear indurated lesion affecting the forehead or scalp. Localized scleroderma itself is an uncommon disorder, with an estimated incidence of approximately 0.3-3 cases per 100,000 individuals annually and a higher frequency in females [1]. The disease can occur in both pediatric and adult populations; however, craniofacial linear variants, such as ECDS, are more commonly reported in children [1,2]. Consequently, cases presenting later in adulthood remain relatively uncommon and may present a diagnostic challenge.

Much of the current understanding of disease behavior and extracutaneous manifestations in ECDS is derived from pediatric cohorts [1-3]. During craniofacial development, lesions may progress and extend into deeper structures, including subcutaneous tissue, bone, and, occasionally, intracranial structures [1,2]. In contrast, adult-onset ECDS is uncommon, and comparative data evaluating differences between early- and late-onset disease remain limited [4]. Existing reports have not demonstrated a clear difference in the rate of cutaneous progression between age groups. However, some authors suggest that the degree of structural deformity may be less pronounced in adult-onset disease because craniofacial skeletal growth has already been completed [1,4]. Neurologic manifestations such as seizures, headaches, and white matter abnormalities have been reported in association with ECDS in both pediatric and adult patients [5-8]. Nevertheless, these complications appear to be described more frequently in early-onset disease, which may reflect longer disease duration and involvement during periods of craniofacial development [1-3]. Accordingly, ongoing neurologic monitoring is recommended for patients with craniofacial ECDS regardless of the age at disease onset. The present case contributes to the limited literature on late-onset disease and highlights the importance of considering ECDS in adults presenting with progressive craniofacial atrophy.

Although localized scleroderma has traditionally been regarded as confined to cutaneous tissues, multiple studies have shown that the linear craniofacial variant can extend deeper, affecting fat, muscle, bone, and the central nervous system [1,2,9]. Neurologic involvement has included headaches, focal seizures, cognitive impairment, and status epilepticus, often correlating with ipsilateral white matter abnormalities [5-7]. More recent evidence from advanced neuroimaging has demonstrated subclinical findings, including perfusion deficits and metabolic changes in affected brain regions [8]. These observations support the concept that ECDS may represent a neurocutaneous inflammatory condition rather than a purely dermatologic disorder. The absence of neurologic features in our patient at presentation is reassuring, but continued surveillance was warranted given the location of the disease and the possibility of delayed intracranial involvement. Because neurologic complications may occur during the disease course, several authors recommend neuroimaging evaluation when craniofacial linear scleroderma is diagnosed [1,2,5]. Magnetic resonance imaging (MRI), particularly with T2 and FLAIR sequences, is considered the most sensitive modality for detecting intracranial abnormalities associated with ECDS. In the present case, MRI was not performed because the patient did not exhibit neurologic symptoms and declined further imaging due to financial constraints.

The pathogenesis of ECDS involves immune-mediated vascular injury, which contributes to chronic tissue ischemia and progressive fibrosis of the skin and deeper structures [1-3]. Inflammatory activation of endothelial cells and subsequent fibroblast stimulation lead to excessive collagen deposition and dermal sclerosis, which clinically manifest as skin induration and soft-tissue atrophy [1]. Craniofacial variants may extend to bone and neural tissue, supported by neuroimaging and biopsy studies that have identified ipsilateral white matter changes and inflammatory infiltrates linked to the neurovascular mechanism of disease [5,8]. This provides a biologic rationale for the need for neurologic monitoring throughout the course of craniofacial ECDS, even when initial symptoms are limited to the skin.

Extracutaneous manifestations are not limited to the nervous system. Case reports have described intraoral changes, mandibular bone remodeling, and dental malalignment, particularly in pediatric patients [9,10]. Classically, ECDS presents as a unilateral linear lesion affecting one side of the face. However, atypical presentations, including extension beyond the initial distribution or mild involvement across the midline, have occasionally been described [1,9]. The mild contralateral extension observed in our patient, therefore, represents an atypical but possible manifestation within the spectrum of craniofacial linear scleroderma. Given the involvement of the nasal and frontal regions, ongoing multidisciplinary follow-up is appropriate, including consideration of dental and maxillofacial evaluation if facial asymmetry progresses.

Diagnostically, localized scleroderma with facial involvement can be mistaken for vasculitis, deep fungal infections, or Hansen disease. The linear configuration of the lesion in our patient, along with negative infectious testing, normal vasculitic markers, and characteristic histopathology, supported a diagnosis of ECDS. Positive anti-histone antibodies have been reported in localized scleroderma but lack diagnostic specificity and are considered more reflective of autoimmune activation [1].

Early initiation of systemic immunosuppressive therapy has been recommended for craniofacial variants to limit irreversible tissue damage and reduce the likelihood of deeper organ involvement [1,2]. Systemic corticosteroids are commonly used during the active inflammatory phase to suppress disease progression, often in combination with steroid-sparing agents such as methotrexate, which is considered first-line immunomodulatory therapy for moderate to severe localized scleroderma [1-3]. Adjunctive therapies, including phototherapy, particularly psoralen plus ultraviolet A (PUVA), have also been reported to improve cutaneous sclerosis in selected cases [1,9]. The management approach in this case included initiation of systemic corticosteroids and referral to dermatology and rheumatology specialists for consideration of steroid-sparing immunomodulatory therapy and phototherapy, aligning with current best practice guidance reported in the literature [1,2,8,9]. However, the patient returned to her home country before long-term immunomodulatory therapy could be initiated at our institution; therefore, the specific agent and dosing regimen could not be documented.

This report has several limitations. Magnetic resonance imaging was not performed because the patient did not exhibit neurologic symptoms and declined further imaging due to financial constraints. Histopathological images were not available for inclusion because the original biopsy slides were taken by the patient when she returned to her home country. Original radiological images, including chest X-ray and contrast-enhanced CT, were not available for retrieval, which limited the ability to provide visual diagnostic correlation. Additionally, long-term follow-up could not be obtained because the patient traveled abroad shortly after diagnosis, preventing assessment of treatment response and disease progression.

Despite these limitations, this report highlights several important clinical aspects, including the rare late-onset presentation of ECDS in an elderly patient and the diagnostic challenges associated with atypical craniofacial involvement. It reinforces the importance of physician awareness when evaluating progressive frontofacial changes in adults and highlights the need for timely diagnosis and coordinated long-term follow-up to prevent cosmetic and functional complications. The detailed clinical description and exclusion of alternative diagnoses contribute to the limited literature on adult-onset ECDS.

## Conclusions

ECDS typically presents during childhood, and late-onset disease is uncommon. This case emphasizes the importance of considering linear scleroderma in the differential diagnosis of progressive frontofacial atrophy in adults, particularly in atypical presentations with extension beyond the usual unilateral distribution. Early recognition and prompt initiation of systemic therapy are crucial to limit permanent disfigurement and reduce the risk of neurologic and other extracutaneous complications. Close multidisciplinary follow-up remains essential even in the absence of initial systemic involvement.
